# The importance of behavioral data to identify online fake reviews for tourism businesses: a systematic review

**DOI:** 10.7717/peerj-cs.219

**Published:** 2019-09-23

**Authors:** Ana Reyes-Menendez, Jose Ramon Saura, Ferrão Filipe

**Affiliations:** 1 Department of Business Economics, Rey Juan Carlos University, Madrid, Spain; 2 Vice-Rector Universidade Portucalense, Universidade Portucalense Infante D. Henrique, Porto, Portugal

**Keywords:** Online reviews, Fake reviews, Consumer behavior, Algorithms, Tourism

## Abstract

In the last several decades, electronic word of mouth (eWOM) has been widely used by consumers on different digital platforms to gather feedback about products and services from previous customer behavior. However, this useful information is getting blurred by fake reviews—i.e., reviews that were created artificially and are thus not representative of real customer opinions. The present study aims to thoroughly investigate the phenomenon of fake online reviews in the tourism sector on social networking and online reviews sites. To this end, we conducted a systematic review of the literature on fake reviews for tourism businesses. Our focus was on previous studies that addressed the following two main topics: (i) tourism (ii) fake reviews. Scientific databases were used to collect relevant literature. The search terms “tourism” and “fake reviews” were applied. The database of Web of Science produced a total of 124 articles and, after the application of different filters following the PRISMA 2009 Flow diagram, the process resulted in the selection of 17 studies. Our results demonstrate that (i) the analysis of fake reviews is interdisciplinary, ranging from Computer Science to Business and Management, (ii) the methods are based on algorithms and sentiment analysis, while other methodologies are rarely used; and (iii) the current and future state of fraudulent detection is based on emotional approaches, semantic analysis and new technologies such as Blockchain. This study also provides helpful strategies to counteract the ubiquity of fake reviews for tourism businesses.

## Introduction

In the last four decades, the continuously growing sector of tourism has been supported by the development of information and communication technologies (ICT) (Papathanassis & Buhalis, 2007; Buhalis & Law, 2008). In the 21st century, the digital revolution in social sciences and tourism should be taken into account, as it is one of the important factors that make the tourism industry competitive (Moutinho, Ballantyne & Rate, 2011; Saura & Bennett, 2019).

Nowadays, consumers use different social platforms, such as social networking sites (SNS), consumer review sites, blogs, and social communities in order to communicate and share their purchase experiences and behavior regarding for products and brands with other consumers (Cheung & Thadani, 2012; Chew, Metheney & Teague, 2017; Hubert et al., 2017).

The continuously developing technologies and the wide spread use of the Internet in several industries have empowered the evolution from traditional word-of-mouth to electronic word-of-mouth (eWOM) (Gottschalk & Mafael, 2017; Manes & Tchetchik, 2018). eWOM is embodied in online reviews that customers write for other customers. The content of online reviews depends on the experience that these specific customers have with purchased products or services (Munar & Jacobsen, 2014; Saura, Reyes-Menendez & Alvarez-Alonso, 2018a). This fact has an important consequence for businesses, as there is a power shift from companies to consumers (Hennig-Thurau, Walsh & Walsh, 2003; Reyes-Menendez et al., 2018). The abovementioned power shift is particularly important in certain industries, such as the tourism industry where customers pay close attention to the opinion of previous travelers (Papathanassis & Knolle, 2011). For that reason, online reviews are a powerful communication tool for tourism businesses. Tourism businesses are companies such as hotels, ancillary services, transportation companies and restaurants (Riegner, 2007; Reyes-Menendez, Saura & Palos-Sánchez, 2018b).

With the growth of the Internet, the number of online reviews has increased as well, exerting a significant influence on customers’ purchase decision making (Bennett, Yábar & Saura, 2017). The growing relevance of this type of communication is particularly important on social platforms where it takes the form of online reviews. However, what happens when this information does not represent the objective reality? Are companies, rather than real consumers, writing these reviews?

In 2019, the Federal Trade Commission (FTC) denounced, for the first time, a company that advertised products to lose weight on Amazon for writing false reviews on this platform. In a press interview, the director of FTC, Andrew Smith, said that false reviews adversely affect both consumers and companies, as they represent a breach of market norms. For their part, Amazon representatives declared that they would take legal action against those fake reviews and invest significant economic and human resources to ensure that the reviews of the products presented on their platform are true and up-to-date (Shu, 2019).

**Figure 1:**
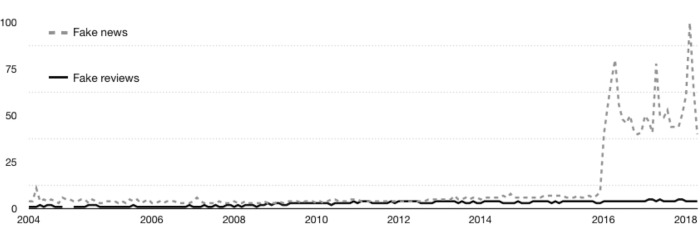
Evolution of searches about fake news and fake reviews. Source: Google Trends (2018).

**Figure 2:**
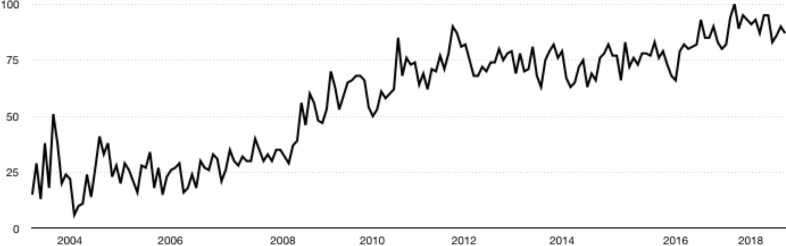
Evolution of searches about fake reviews. Source: Google Trends (2018).

Overall, consumers are becoming increasingly aware that many of the reviews on social network sites are fraudulent. To show this trend in numbers, following Önder & Ulrich Gunter (2016) we first made a search with Google Trends, a tool used previously (Önder, Irem & Ulrich Gunter, 2016) to identify past search trends in Google on various topics of interest. The results are shown in Figs. 1 and 2.

In Fig. 1, the searches on “fake reviews” made by users on Google are shown with a solid line, while searches on “fake news” are shown with a dotted line. As can be seen in Fig. 1, the number of searches on both topics has increased from 2004 to 2018. However, the increase is dramatically more pronounced for the searches related to “fake news”. The dynamics of the growth in the number of searches on “fake reviews” only is shown in Fig. 2. As can be seen in Fig. 2, the number of searches on “fake reviews” has steadily increased throughout the period 2004–2018, and this number continues to grow.

In the next step, we checked the importance of the topic of fake reviews for the scientific community. This was done with a search in Web of Science (WOS), a scientific database that indexes scientific articles.

Similarly to the results obtained with Google Trends, the WOS findings suggest that, throughout 2004–2018, there has been a considerable growth in the number of articles published on the issues of fake news and fake online reviews. Furthermore, again consistent with the results of using Google Trends, the scientific community has been more interested in the topic of fake news, as we found 248 papers that include the term “fake news” in the title, while there were only 48 papers with the term “fake reviews” in the title.

**Figure 3:**
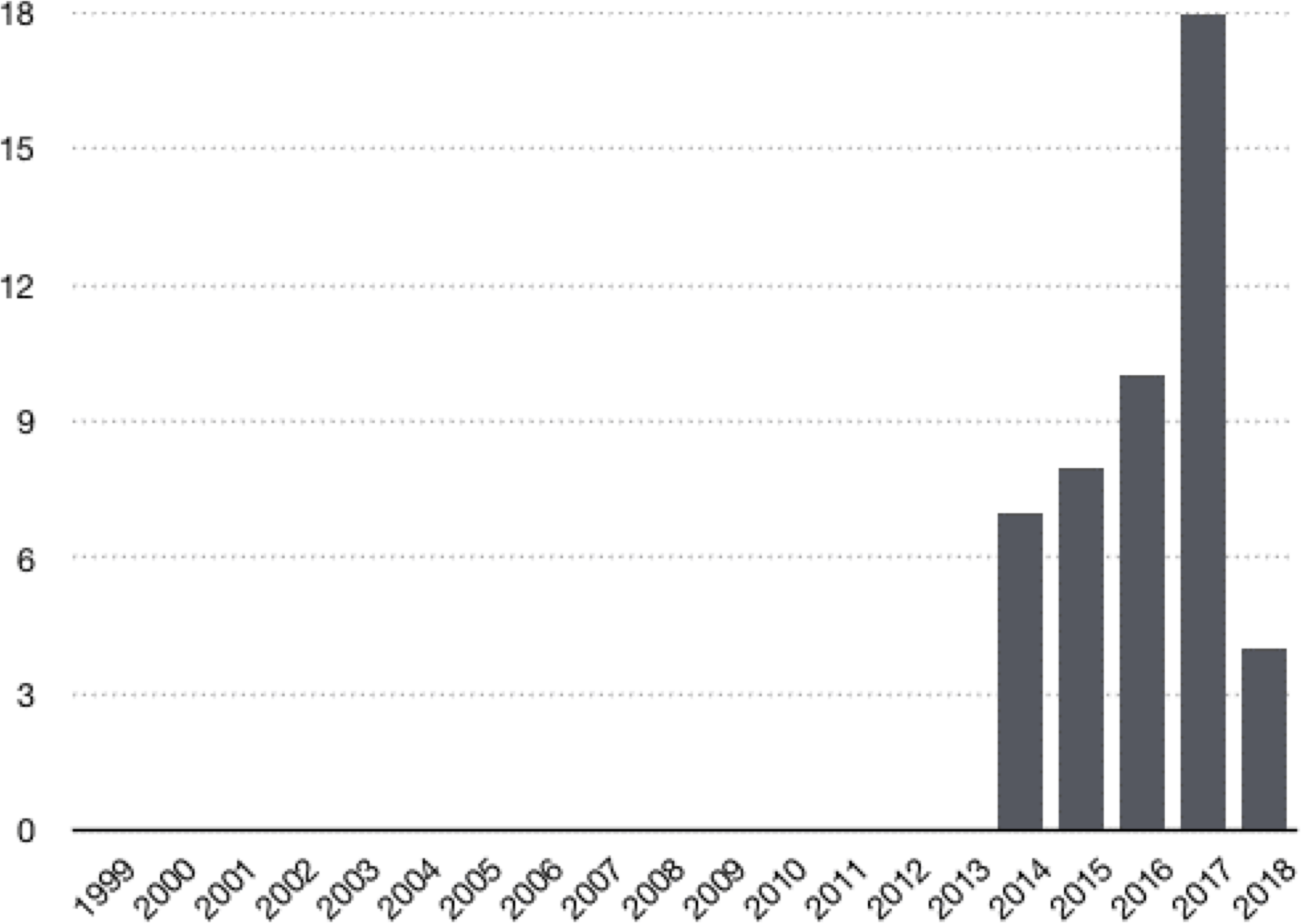
Number of citations to articles by year. Source: Google Trends (2018).

Figure 3 shows the dynamics in the number of citations to articles addressing fake online reviews throughout the period 2004–2018. As can be seen in Fig. 3, the first citations appeared in 2013. However, it was not until 2014 that scientific interest in fake online reviews skyrocketed, and it continues to grow today. Of note, according to the publication terms of the journals included in the JCR (Journal Citation Report) Index to which Web of Science (WOS) belongs, the publications that appeared in 2018 will begin to get cited in the next months or years. This explains why the publications from 2018 do not follow the growth trend as compared to the previous years.

The results shown in Figs. 1–3 underscore the importance of the topic of fake online reviews for consumers, companies, and the scientific community. Accordingly, in the present paper, our major goal is to identify directions of current research to address the problem of fake reviews on tourism platforms.

The remainder of this paper is structured as follows. After a brief literature review in ‘Literature Review’, we present the methodology used in the present study in ‘Methodology’. Results are reported in ‘Exploratory analysis of results’. The paper concludes with a discussion of implications of our findings (‘Implications’) and general conclusions (‘Conclusions’).

## Literature Review

Over the last years, many studies have investigated the impact of online reviews on consumer purchase behavior and decision making (Chevalier & Mayzlin, 2003; Riegner, 2007; Gretzel & Yoo, 2008). A strong influence of online reviews has also been highlighted in numerous industrial statistic reports (e.g., Reyes-Menendez et al., 2018).

Electronic word-of-mouth has become an important concept for tourism businesses (Papathanassis & Knolle, 2011). According to Litvin, Goldsmith & Pan (2008) and Pai et al. (2013), eWOM is the most important source of information that drives consumer purchase behavior in the hospitality and tourism services sectors. In the last several decades, advances in information and communication technologies (ICTs) have transformed both travelers’ behavior and the tourism industry (Buhalis & Law, 2008). Nowadays, the number of travelers who access the Internet to book hotel rooms via third-party intermediaries is continuously increasing (Luo, Chen & Zheng, 2016). Furthermore, several studies demonstrated that about two-thirds of customers prefer to read online consumer reviews about a hotel, rather rely on the hotel’s own descriptions. Such online reviews are visited by hundreds of millions of potential hotel visitors every year (Reyes-Menendez, Saura & Martinez-Navalon, 2019)

Therefore, in order to obtain a better understanding of the continuously increasing impact of eWOM on different social platforms and its effect on the decision making and behavior of hotel consumers, reviews on online travel sites and social networking sites should be taken into account (Saura, Rodriguez Herráez & Reyes-Menendez, 2019). Yet, a recently emerging issue with online reviews is that some online reviews are fake. Although most online platforms have their own false review detection algorithms (Cheng, Tseng & Chung, 2017), these algorithms are sometimes limited in scope and filter only 16% of published fake reviews (Luca & Zervas, 2016). Therefore, there is a clear need to improve the existing algorithms and elaborate new approaches. Many studies have sought to do just that (e.g., Elmurngi & Gherbi, 2017; Munzel, 2016; Zhang et al., 2016). To this end, various methodologies have been used, some of which will be discussed in the remainder of this paper.

## Methodology

Following Kazakov & Predvoditeleva (2015), in the present study, we aimed to provide an overview of previous research on the state of art of online fake reviews in tourism social networking sites. We focused on the analysis of users’ ability to detect real or fake reviews. To this end, we critically examined the available literature on tourism fake reviews and behavioral approaches to analyze and identify them for tourism businesses. The systematic literature review focused on the following two main topics: (i) fake reviews; (ii) tourism. Following Sherman et al. (2005) and Banerjee, Chua & Kim (2015), we used a randomized controlled process to select the main topics and consequent search terms “fake reviews” and “tourism”.

**Figure 4:**
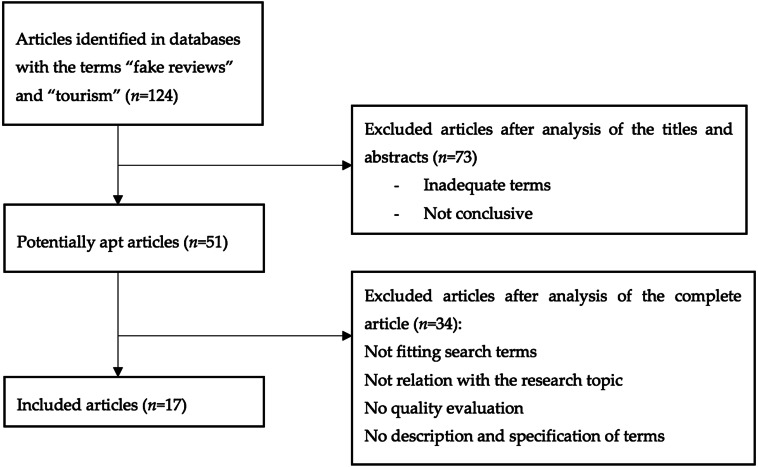
PRISMA 2009 Flow Diagram.

The scientific databases of Scopus, PubMed, PsyINFO, ScienceDirect, and Web of Science were used to collect relevant studies on the issue at stake. Of note, when performing the search by “Title” in the scientific database Web of Science, only one article met the aforementioned search requirement, that both “fake reviews AND tourism” were contained in the title. Therefore, following Saura, Palos-Sánchez & Suárez (2017), we included the articles that were initially obtained as a result of the search, prioritizing those that dealt with reviews, even if they were not specifically focused on tourism platforms. We reasoned that the insights reported in these studies could be extended to address the problem of false reviews on tourism platforms. The search yielded a total of 124 articles; after different filters were applied (see Fig. 4), a total of 17 studies were selected for further analysis. The Boolean operator AND was applied to optimize the results. All articles were analyzed by reading the titles and abstracts and selecting the ones which met the inclusion criteria. Next, we analyzed the 17 selected papers. The data were collected in June 2018 using AMSTAR (2017), a tool initially designed to assess the quality of articles based on their abstracts (Shea et al., 2009). In this way, we ensured that only high-quality studies were included in the dataset. In the process of article selection, we also followed the recommendations formulated by Van den Bosch & Ode Sang (2017). These recommendations include keyword search in several databases, predefined inclusion criteria, and data extraction based on selected keywords.

To this end, following Saura, Palos-Sánchez & Suárez (2017), we used the Preferred Reporting Items for Systematic reviews and Meta-Analyses (PRISMA) 2009 Flow Diagram. This method, introduced by Moher et al. (2009), provides guidelines to develop Systematic Reviews and Meta-Analyses that include conceptual and practical advances in the science of systematic reviews. One of the phases of the PRISMA Flow Diagram is discarding the articles that have inadequate or inconclusive terms. The terms considered as inadequate or inconclusive are those that *a priori* may correspond to the keywords; however, when reading the article in depth, it is observed that they are not within the scope of the investigation. These terms can be misleading, as in the case of reviews that may be either tourist reviews or peer reviews.

Our aim was to achieve the highest possible amount of evidence in the results based on high-quality studies. Some of the variables used in AMSTAR to evaluate the quality of the systematic review were (i) the relationship of the research question to the criteria included in the study; (ii) the extraction of data from at least two independent researchers; (iii) the quality of the literature review, (iii) identification and definition of concepts; and (iv) the quality of the conclusions stated in the study.

## Exploratory Analysis of Results

The Systematic Literature Review (RSL) was proposed by Saura, Palos-Sánchez & Suárez (2017) and Bassett (2015) as a development tool to carry out an exploratory analysis of previously reported results. Such a literature review is used to evaluate researchers’ interest in a specific topic (Saura, Reyes-Menendez & Palos-Sanchez, 2018b). A literature review is an exploratory methodology and consists of collecting and re-analyzing available findings. A literature review usually includes both primary and secondary sources.

Luo & Zhong (2015) and Comerio & Strozzi (2018) conducted literature reviews and applied exploratory analysis specifically for tourism businesses, while Huete-Alcocer (2017) focused on the transformation of the traditional word-of-mouth into electronic word-of-mouth, and the behavioral implications of this transition for the tourism industry.

A summary of the studies selected for further analysis in the present review is shown in Table 1. Table 1 presents the authors of relevant studies, as well as the main contents of those studies. Based on this information, we categorized the reviewed studies in several groups. For instance, the studies that can be applied to all sectors were categorized as “All Industries”. A study classified into the category of “E-Commerce” was included in the analysis since, for example, TripAdvisor is considered an E-commerce platform in the tourism sector. Studies on restaurants were categorized into the “Restaurant—Hospitality Industry” group. Finally, articles on hotels and other tourist services were grouped into the “Tourism” category.

**Table 1:** Previous studies on fake online reviews in the tourism industry.

**Authors**	**Study description**	**Study industry**
Banerjee & Chua (2014)	This study proposes several algorithms of identification of false reviews. Attention is paid to linguistic aspects of comprehension, level of detail, writing form, and cognitive indicators	Tourism
Banerjee et al. (2015)	This study investigates false reviews published on TripAdvisor. After completing a survey, users are invited to write fake hotel reviews	Tourism
Cardoso, Silva & Almeida (2018)	This paper is an exhaustive review of the content analysis methods of false review detection. To this end, the authors develop experiments based on Hotels	Tourism
Chang et al. (2015)	In this study, a rumor model is used to detect false reviews on the TripAdvisor platform based on the following three characteristics of the content: important attribute words, quantifiers, and the ratio of names and verbs. The proposed model reduces the possibility of obtaining false reviews	Tourism
Deng & Chen (2014)	This study focuses on the development of an algorithm, based on sentiment analysis, to identify false reviews of restaurants. The results demonstrate that the proposed algorithm has the predictive capacity of over 70%	Tourism
Hunt (2015)	This study focuses on the legal aspect of fake reviews and argues for the adoption of specific laws to prohibit the publication of false reviews	Tourism
Lappas, Sabnis & Valkanas (2016a) and Lappas, Sabnis & Valkanas (2016b)	The analysis is based on over 2.3 million comments from 4,709 hotels in 17 cities to understand the impact of false reviews on the visibility of establishments. The results suggest that, with only 50 false reviews in some markets, competitors can be overtaken in terms of visibility	Tourism
Li, Feng & Zhang (2016)	Based on the density of the reviews, as well as their semantic aspects and emotional aspects, this study creates an algorithm for false review detection based on review content applicable to the tourism industry	Tourism
Munzel (2016)	This study analyzes published reviews and rejected reviews taking into account the information about the author, age, and stars the user has been given in recently published reviews. The results emphasize the importance of the previous history of users who publish reviews for false review detection	Tourism
Chen, Guo & Deng (2014)	This study proposes an algorithm based on sentiment analysis to identify false reviews in restaurants. The results demonstrate that the proposed algorithm has the predictive capacity of 74%	Restaurants - Hospitality Industry
Li et al. (2014)	This study focuses on the Dianping, China’s largest restaurant review platform, and analyzes the dependencies among reviews, users, and IP addresses using an algorithm called Multi-typed Heterogeneous Collective Classification (MHCC), and then extends it to Collective Positive and Unlabeled learning (CPU)	Restaurants - Hospitality Industry
Li et al. (2018)	In this study, the Louvain community detection method is used to study online communities. The results suggest that false reviews predominate in profiles with low scores, and that the more followers a community has, the greater the number of false reviews	Restaurants - Hospitality Industry
Luca & Zervas (2016)	This study analyzes the reviews published on the Yelp site. The results demonstrate that only 16% of the reviews are filtered (those that more extreme, either positively or negatively). The restaurants that usually publish false reviews are those with fewer comments or negative comments. Restaurant chains usually publish fewer false reviews. Finally, more competitive restaurants are more likely to get false reviews	Restaurants - Hospitality Industry
Elmurngi & Gherbi (2017)	In this study, textual classification and sentiment analysis are used to identify false reviews in E-commerce. Four rating sentiment classification algorithms are compared: Naïve Bayes (NB), Support Vector Machine (SVM), K-Nearest Neighbors (KNN-IBK), and Decision Tree (DT-J48). The results show that algorithms can effectively predict false reviews	E-Commerce
Lin et al. (2014)	This study proposes a new approach to identifying false reviews that is based on the content of the reviews and the behavior of the users. The results show that the proposed approach is more precise and accurate than current algorithms	All Industries
Ramalingam & Chinnaiah (2018)	This study reviews the latest algorithms of false profile detection in social networks	All Industries
Zhang et al. (2016)	This study analyzes non-verbal characteristics of users who write false reviews to create a predictive algorithm of detection of false reviews. The algorithm can complement the traditional method of detection of false reviews	All Industries

The studies summarized in Table 1 demonstrate the growing interest in the concept of fake reviews and social networking sites, particularly in the hospitality and tourism industries. Some of these studies (e.g., Banerjee & Chua, 2014; Banerjee et al., 2015; Cardoso, Silva & Almeida, 2018; Chang et al., 2015; Deng & Chen, 2014; Hunt, 2015; Lappas, Sabnis & Valkanas, 2016a; Lappas, Sabnis & Valkanas, 2016b; Li, Feng & Zhang, 2016; Munzel, 2016) focus on the Tourism industry category, while others fall into the hospitality industry category (Chen, Guo & Deng, 2014; Li et al., 2014; Li et al., 2018; Luca & Zervas, 2016). Some works (Lin et al., 2014; Zhang et al., 2016; Ramalingam & Chinnaiah, 2018) were included as part of the analysis because their results can be implemented in every industry that allows consumers to write reviews, including the tourism industry. Elmurngi & Gherbi (2018) analyzed false reviews in E-commerce, considering that TripAdvisor is the most important e-commerce platform in the hospitality industry; therefore, this study might be of interest to the present study (Reyes-Menendez, Saura & Alvarez-Alonso, 2018a).

The interest in the concept of fake reviews is underpinned by two factors. First, as demonstrated in several studies, the currently available algorithms of false review detection remain largely ineffective. For instance, Luca & Zervas’ (2016) results demonstrate that only 16% of the false reviews are filtered on the Yelp platform—particularly, those that have more extreme content, either positive or negative; this suggests that the remaining 84% of reviews are not filtered and may be false. Second, as demonstrated in several studies, false reviews negatively impact companies’ visibility. For instance, Lappas, Sabnis & Valkanas (2016a) and Lappas, Sabnis & Valkanas (2016b) report that, with only 50 false reviews in some markets, competitors can be overtaken in terms of visibility. In this sense, Hunt (2015) focuses on the example of the UK Advertising Standards Authority that found against the tourism social platform TripAdvisor. These figures explain the concern of establishments in the tourism sector about the phenomenon of false reviews.

In what follows, we review the methodologies used in previous research on false reviews in the tourism industry. Particular attention is paid to the unit of analysis focused on in previous studies and the behavioral approach to the analysis and identification of online fake reviews in tourism.

### Methodologies used in previous research

First, most studies in the present systematic review of the literature focus on analysis of the algorithms of false review detection and their improvement. In these studies, large amounts of data from social communities such as TripAdvisor or Yelp are typically used (Chang et al., 2015; Li et al., 2014). The second most used methodology is sentiment analysis, focusing on the emotional aspects and feelings expressed in written reviews. In this methodology, comments are classified as positive, negative or neutral according to the words contained in them (Chen, Guo & Deng, 2014; Deng & Chen, 2014; Elmurngi & Gherbi, 2017). The third direction of research comprises other methodologies that aim to either discover new knowledge to be implemented in false review detection for tourism businesses (Hunt, 2015) or perform the analysis of legal aspects and measures that countries like the United Kingdom or Australia take to counteract false reviews (Ramalingam & Chinnaiah, 2018). Table 2 provides a classification of the studies reviewed in the present study into the aforementioned three groups.

**Table 2:** Classification of previous studies according to their methodology.

**Authors**	**Algorithms**	**Sentiment analysis**	**Other methodologies**
Banerjee & Chua (2014)	√		
Banerjee et al. (2015)	√		
Cardoso, Silva & Almeida (2018)	√		
Chang et al. (2015)	√		
Li et al. (2014)	√		
Li, Feng & Zhang (2016)	√		
Li et al. (2018)	√		
Luca & Zervas (2016)	√		
Lappas, Sabnis & Valkanas (2016a) and Lappas, Sabnis & Valkanas (2016b)		√	
Chen, Guo & Deng (2014)		√	
Deng & Chen (2014)		√	
Elmurngi & Gherbi (2017)		√	
Lin et al. (2014)		√	
Zhang et al. (2016)		√	
Hunt (2015)			√
Munzel (2016)			√
Ramalingam & Chinnaiah (2018)			√

### Unit of analysis

The studies reviewed in this paper approach the phenomenon of false reviews differently. For some studies (Li et al., 2014; Lin et al., 2014; Ramalingam & Chinnaiah, 2018) the major units of analysis are profiles of users who write reviews. These studies seek patterns that would help better identify profiles that are more likely to generate false reviews.

For other studies, the major unit of analysis is the content of online reviews. Here, the studies tend to focus on two types of content. On the one hand, some studies focus on the textual content of reviews, i.e., on their linguistic aspects, such as the ratio of nouns to verbs, the type of words or the attributes used to write false reviews (Banerjee et al., 2015; Cardoso, Silva & Almeida, 2018; Chang et al., 2015; Chen, Guo & Deng, 2014; Deng & Chen, 2014; Elmurngi & Gherbi, 2017; Lappas, Sabnis & Valkanas, 2016a; Lappas, Sabnis & Valkanas, 2016b). On the other hand, there are studies that prioritize detecting behavioral and emotional characteristics of users who write false reviews (Banerjee & Chua, 2014; Li, Feng & Zhang, 2016; Li et al., 2018; Luca & Zervas, 2016; Zhang et al., 2016). Munzel (2016) focuses the research on both the textual and the behavioral aspects. At the same time, Hunt (2015) does a review of the legal aspects that concern fake reviews, such that the unit of analysis is neither user profile nor content. In Table 3, the reviewed studies are classified into the aforementioned three groups.

**Table 3:** Classification of previous studies according to their unit of analysis.

**Authors**	**User profile**	**Content**	
		**Textual**	**Behavioral**
Li et al. (2014)	√	–	–
Lin et al. (2014)	√	–	–
Ramalingam & Chinnaiah (2018)	√	–	–
Banerjee et al. (2015)	–	√	–
Cardoso, Silva & Almeida (2018)	–	√	–
Chang et al. (2015)	–	√	–
Chen, Guo & Deng (2014)	–	√	–
Deng & Chen (2014)	–	√	–
Elmurngi & Gherbi (2017)	–	√	–
Lappas, Sabnis & Valkanas (2016a) and Lappas, Sabnis & Valkanas (2016b)	–	√	–
Munzel (2016)	–	√	√
Banerjee & Chua (2014)	–	–	√
Li, Feng & Zhang (2016)	–	–	√
Li et al. (2018)	–	–	√
Luca & Zervas (2016)	–	–	√
Zhang et al. (2016)	–	–	√
Hunt (2015)	–	–	–

### Scientometric analysis

In the next step, in order to gain a better understanding of which areas of research focused more on false reviews, scientometric analysis was performed. Here, we followed a previous study by Saura, Palos-Sánchez & Suárez (2017).

A scientometric analysis is the quantitative and qualitative analysis of science and scientific outcomes. This concept was first created by Price (1963) and has been widely used as a complementary analysis of systematic literature reviews (Saura, Palos-Sánchez & Suárez, 2017) or as the major topic of the study (Kullenberg & Kasperowski, 2016). In their work, Kollwitz & Papathanassis (2011) highlighted the importance of using qualitative methods, such as Delphi techniques for the tourism industry, and Walle (1997) supported using a combination of qualitative with quantitative analysis in the tourism sector.

In Table 4, the results of both qualitative and quantitative analysis are summarized with respect to the Author, Journal indexed in JCR ranking and its Category and Quartile. The Author field is included to trace the analysis conducted by the authors throughout the article. The Quartile and Category reflect all the categories that the journal has according to its Web of Science classification and, in case the Quartile was different for different Categories, that is also reflected in the table.

**Table 4:** Scientometric classification.

**Author**	**Journal**	**Quartile**	**Category**
Banerjee & Chua (2014)	9th International Conference on Digital Information Management (ICDIM)	–	Computer Science, Information Systems; Computer Science, Theory & Methods; Engineering, Electrical & Electronic
Banerjee et al. (2015)	9th International Conference on Ubiquitous Information Management and Communication (ACM IMCOM)	–	Computer Science, Theory & Methods
Cardoso, Silva & Almeida (2018)	Neurocomputing	Q1	Computer Science; Artificial Intelligence
Chang et al. (2015)	Lecture Notes in Computer Science	Q4	Computer Science, Theory & Methods
Chen, Guo & Deng (2014)	Lecture Notes in Computer Science	Q4	Computer Science
Deng & Chen (2014)	Lecture Notes in Computer Science	Q4	Computer Science, Theory & Methods
Elmurngi & Gherbi (2017)	7th International Conference on Innovative Computing Technology (INTECH)	–	Computer Science, Hardware & Architecture; Computer Science, Software Engineering; Computer Science, Theory & Methods
Hunt (2015)	Computer Law and Security Review	Q2	Law
Lappas, Sabnis & Valkanas (2016a) and Lappas, Sabnis & Valkanas (2016b)	Information Systems Research	Q2	Management Information Science & Library Science
Li, Feng & Zhang (2016)	2016 3RD International Conference on Information Science and Control Engineering (ICISCE)	–	Automation & Control Systems, Computer Science, Theory & Methods
Li et al. (2014)	IEEE International Conference on Data Mining (ICDM)	–	Computer Science, Artificial Intelligence; Computer Science, Information Systems
Li et al. (2018)	China Communications	Q3	Telecommunications
Lin et al. (2014)	2014 Proceedings of the IEEE/ACM International Conference on Advances in Social Networks Analysis and Mining (ASONAM 2014)	–	Computer Science, Information Systems; Computer Science, Theory & Methods
Luca & Zervas (2016)	Management Science	Q1	Management Operations Research & Management Science
Munzel (2016)	Journal of Retailing and Customer Services	Q2	Business
Ramalingam & Chinnaiah (2018)	Computers and Electronical Engineering	Q2	Computer Science; Engineering, Electrical & Electronic
		Q3	Computer Science, Interdisciplinary
Zhang et al. (2016)	Journal of Management Information Systems	Q2	Management Computer Science, Information Systems
		Q1	Information Science & Library Science

The discipline with the highest number of publications was Computer Science as can be seen in Table 4. There are a total of 11 publications that belong to the category of Computer Science and six that fall into the category of Computer Science Theory & Methods. The next category is Information Systems which has four publications. Other disciplines include Management (three publications), and Operations Research, Management Science, and Business (one publication each). This indicates that fake reviews are of interest to both computer scientists who develop the algorithms of false review detection and the management of companies and businesses that is willing to improve the detection of fake reviews that undermine the credibility of these platforms. All reviewed studies come from the disciplines of Artificial Intelligence, Automation & Control Systems, Business, Computer Science, Computer Science Theory & Methods, Engineering Electrical & Electronic, Hardware & Architecture, Information Systems, Information Science & Library Science, Interdisciplinary, Software Engineering, Law, Management, Operations Research & Management Science and finally, Telecommunications.

All journals have only published one paper, except for the journal *Lecture Notes in Computer Science* that published three articles (Chen, Guo & Deng, 2014; Deng & Chen, 2014; Chang et al., 2015).

It is also interesting to note that six of the papers that were listed in the database of the Web of Science and for which we could extract the category were published as Conference Proceedings; therefore, their Quartile could not be analyzed.

Regarding the Quartile of the remaining papers, we took the highest rated category in the case when there was more than one category with different Quartiles. Therefore, three of them were Q1, four were Q2, and three were Q4.

## Implications

### Implications for managers

The results of the present study underscore the importance of online reviews for the tourism industry not only on major websites (Papathanassis, 2011), but also on other type of platforms that require managerial attention for proper brand management (Saura, Palos-Sanchez & Reyes-Menendez, 2017).

Nowadays, online review sites and social media websites have become an important source of information for consumers and exert a strong influence on consumer purchase behavior and decision making (Gretzel et al., 2006; Kim, Kandampully & Bilgihan, 2018; Manes & Tchetchik, 2018; Reyes-Menendez et al., 2018). Therefore, efficient collection and analysis of fake online reviews can help companies to remain competitive in this industry (Hennig-Thurau, Walsh & Walsh, 2003; Hu et al., 2011; Lin et al., 2006; Zhang et al., 2016).

The ubiquitous presence of fake online reviews on review and social networking sites creates an asymmetry in the information that consumers get about a company. However, when managers track customer comments, the information asymmetry is reduced (Manes & Tchetchik, 2018).

The growth in the number of fake reviews in the tourism industry and the influence of these references on customer behavior and decision making has driven numerous managers to explore the phenomenon of fake reviews and consider the application of new online content strategies (Li, Feng & Zhang, 2016; Li et al., 2018; Ramalingam & Chinnaiah, 2018; Reyes-Menendez et al., 2018).

Considering that fake reviews have the power to provide more visibility for companies and affect their behavior (Lappas, Sabnis & Valkanas, 2016a; Lappas, Sabnis & Valkanas, 2016b), it is necessary to reinforce the creation of online reviews from real customers, avoid the creation of fake reviews, and develop content strategies to support and promote customers who are willing to write true information for other customers online.

### Implications for researchers

Despite the extensive scientific research on fake reviews that offers some solutions to counteract them, it is important to continue improving the algorithms of false review detection on social platforms in the tourism sector. To this end, researchers engaged in this field of study should have a thorough understanding of the available results.

As demonstrated in the present review, different methods have been used in previous research on false reviews. These include the development of algorithms based on Big Data from the social platforms themselves (e.g., Chang et al., 2015; Li et al., 2014) as well as sentiment analysis of written comments (Chen, Guo & Deng, 2014; Deng & Chen, 2014; Elmurngi & Gherbi, 2018). Finally, there is a group of studies that used other methodological approaches (Hunt, 2015; Ramalingam & Chinnaiah, 2018). Researchers may apply our results to reinforce the theoretical frameworks of their scholarship by correctly choosing a methodological procedure for their research.

It is also important to consider the units of analysis for future research studies in this area. We noted that while some studies focused on user profiles (Li et al., 2014), most extant research focused on the content of reviews. In the latter group of studies, attention was paid either to the texts and their linguistic characteristics (e.g., Banerjee et al., 2015; Cardoso, Silva & Almeida, 2018; Chang et al., 2015) or to the emotions and behavior of users that could be inferred from reviews (e.g., Banerjee & Chua, 2014; Banerjee et al., 2015; Li, Feng & Zhang, 2016; Lin et al., 2014; Zhang et al., 2016).

## Conclusions

This exploratory study has defined the scope and identified recent avenues of research on fake online reviews in the tourism industry. Interestingly, even though consumers might be aware that some comments are fictitious, they still rely on them to make decisions (Manes & Tchetchik, 2018).

However, although many studies have investigated the impact of eWOM in the tourism industry (Mauri & Minazzi, 2013; Vermeulen & Seegers, 2009; Xie et al., 2011), further research on the impact of new approaches is necessary to detect fake online reviews for tourism businesses.

As suggested by our literature review, to counteract this trend, tourism companies must constantly improve their methods of detecting false reviews. These methods are mainly based on algorithms (Banerjee & Chua, 2014; Banerjee et al., 2015), and the improvements are especially based on behavioral approaches (Li, Feng & Zhang, 2016; Zhang et al., 2016). In addition, in order not to lose their visitors’ trust (Ladhari & Michaud, 2015), platforms that allow users to write reviews about their experiences with tourism companies take legal action against false reviews (Hunt, 2015). When a company spots fake reviews, it has the right to take action. TripAdvisor has fought fraudulent reviews in thousands of lawsuits.

The systematic review of the literature undertaken in the present study allows us to make the following three conclusions.

First, our results demonstrate that there is an ever-growing interest, among scientific and consumer communities alike, in the credibility of online reviews in the tourism sector with an interdisciplinary approach. Not only have Computer Science and Information Systems (e.g., Chen, Guo & Deng, 2014; Chang et al., 2015; Li et al., 2014; Zhang et al., 2016) published research about fake reviews but also Management, Business and Operation Research and Management Science (e.g., (Lappas, Sabnis & Valkanas, 2016a; Lappas, Sabnis & Valkanas, 2016b; Luca & Zervas, 2016); (Munzel, 2016). Consumer concern about fake reviews is particularly pronounced (see Figs. 1 and 2).

Second, the results of both scientometric analysis and our literature review suggest that the methods of Computer Science are most frequently used in terms of the development and improvement of the methods to detect false reviews. Most publications included in the literature review (14) fall into the category of algorithms (eight) and sentiment analysis (six), while only a few of them (three) use other methods.

Third, our systematic review of the literature highlights the importance of further development of new methods for identifying false reviews based on the following criteria: (1) those based on emotional approaches; (2) those based on semantic analysis; and (3) those based on new technologies.

Some of these methods are based on a behavioral and emotional analysis of the text of reviews in the tourism sector (Banerjee & Chua, 2014; Li et al., 2018; Luca & Zervas, 2016; Munzel, 2016; Zhang et al., 2016). To this end, sentiment analysis and textual analysis were used (Chen, Guo & Deng, 2014; Deng & Chen, 2014; Elmurngi & Gherbi, 2017; Lappas, Sabnis & Valkanas, 2016a; Lappas, Sabnis & Valkanas, 2016b; Lin et al., 2014; Zhang et al., 2016).

Other methods are focused on the semantic analysis of the reviews (Li, Feng & Zhang, 2016; Luca & Zervas, 2016) and provide guidelines to follow in order to detect fraudulent reviews. Some of these clues are based on extreme comments and ratings, as well as on comments lacking detail or reviews where first-person pronouns (e.g., *I, me*) are widely used to simulate sincerity.

Finally, some methods to improve the detection of fake reviews are mostly supported by new technologies (Li et al., 2014) and present novel solutions for fake review detection. One of these technologies is the Blockchain, which requires proof of payment in order to publish a review.

The limitations of this study relate to the number of studies reviewed and the databases consulted. Although the authors consulted the main scientific databases—Scopus, PubMed, PsyINFO, ScienceDirect and Web of Science—there are more databases available for consultation.

While our review of the literature has highlighted several important issues related to fake reviews in the tourism sector, further research that would perform in-depth analysis of specific aspects presented in this paper is needed. Among such possibilities is using quantitative techniques to measure the impact of fake reviews on social networking sites. Another promising area of future research is studying the behavioral aspects of users who write online reviews for tourism businesses.

##  Supplemental Information

10.7717/peerj-cs.219/supp-1 Supplemental Information 1 PRISMA checklist

## Additional Information and Declarations

### Data Availability

The following information was supplied regarding data availability: This is a review article and does not have raw data.
